# Evaluating the Impact of Enhanced Recovery After Surgery Protocols on Surgical Outcomes Following Bariatric Surgery—A Systematic Review and Meta-analysis of Randomised Clinical Trials

**DOI:** 10.1007/s11695-024-07072-0

**Published:** 2024-01-26

**Authors:** Matthew G. Davey, Noel E. Donlon, Naomi M. Fearon, Helen M. Heneghan, John B. Conneely

**Affiliations:** 1 Royal College of Surgeons in Ireland, 123 St Stephens Green, Dublin 2, Ireland; 2 Department of Surgery, Mater Misericordiae University Hospital, Eccles Street, Dublin 7, Ireland; 3 Surgical Professorial Unit, St. Vincent’s University Hospital, Elm Park, Dublin 4, Ireland

**Keywords:** Bariatric surgery, Enhanced recovery after surgery, ERAS, Patient outcomes

## Abstract

**Background:**

Enhanced recovery after surgery (ERAS) programmes are evidence-based care improvement processes for surgical patients, which are designed to decrease the impact the anticipated negative physiological cascades following surgery.

**Aim:**

To perform a systematic review and meta-analysis of randomised clinical trials (RCTs) to evaluate the impact of ERAS protocols on outcomes following bariatric surgery compared to standard care (SC).

**Methods:**

A systematic review was performed in accordance with PRISMA guidelines. Meta-analysis was performed using Review Manager version 5.4

**Results:**

Six RCTs including 740 patients were included. The mean age was 40.2 years, and mean body mass index was 44.1 kg/m^2^. Overall, 54.1% underwent Roux-en-Y gastric bypass surgery (400/740) and 45.9% sleeve gastrectomy (340/700). Overall, patients randomised to ERAS programmes had a significant reduction in nausea and vomiting (odds ratio (OR): 0.42, 95% confidence interval (CI): 0.19–0.95, *P* = 0.040), intraoperative time (mean difference (MD): 5.40, 95% CI: 3.05–7.77, *P* < 0.001), time to mobilisation (MD: − 7.78, 95% CI: − 5.46 to − 2.10, *P* < 0.001), intensive care unit stay (ICUS) (MD: 0.70, 95% CI: 0.13–1.27, *P* = 0.020), total hospital stay (THS) (MD: − 0.42, 95% CI: − 0.69 to − 0.16, *P* = 0.002), and functional hospital stay (FHS) (MD: − 0.60, 95% CI: − 0.98 to − 0.22, *P* = 0.002) compared to those who received SC.

**Conclusion:**

ERAS programmes reduce postoperative nausea and vomiting, intraoperative time, time to mobilisation, ICUS, THS, and FHS compared to those who received SC. Accordingly, ERAS should be implemented, where feasible, for patients indicated to undergo bariatric surgery.

**Trial registration** International Prospective Register of Systematic Reviews (PROSPERO – CRD42023434492.

**Graphical Abstract:**

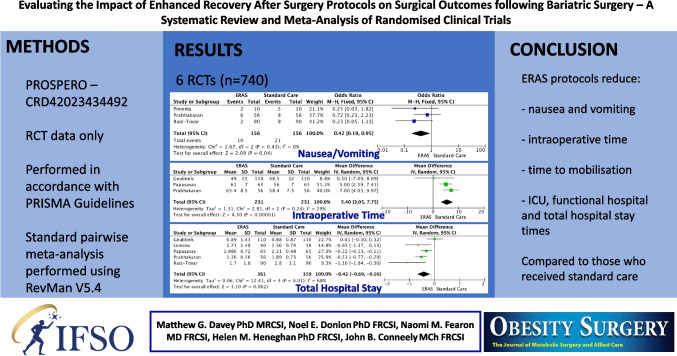

## Introduction

In the western world, the incidence of obesity has increased nearly twofold over the past four decades [1], generating perceptions that the prevalence has reached ‘epidemic proportions’ [2]. Furthermore, it is now estimated that 70% of the adult population are now considered to live with overweight or obesity [1, 3, 4]. Medical therapies, such as glucagon-like peptide-1 receptor agonists, have demonstrated promise in achieving substantial weight loss in patients with obesity [5], as well as significant cardiovascular improvements; however, the response to medication is variable and a significant group of patients do not tolerate the medication due to side effects [6]. The results are impressive, albeit short term, and the treatment is likely required long term for maintenance of effect.

Surgical treatment of obesity remains the most effective and durable option for both significant weight loss and control or prevention of metabolic diseases such as type 2 diabetes mellitus (T2DM) [7, 8] and prevention of a range of malignancies [9, 10]. Moreover, patients who undergo bariatric surgery have significant improvements in their all-cause mortality rates and overall life expectancy compared to those who do not undergo surgery [11]. Accordingly, the pragmatism of using bariatric surgery to improve and optimise patient health has come into vogue, which has subsequently been reciprocated in the dramatic increase in the number of such procedures being performed across Europe and the USA in recent years [12, 13].

Postoperative outcomes following bariatric surgery have improved significantly in recent times [14]. This is likely due to widespread adoption of minimally invasive approaches [15, 16], the centralisation of bariatric surgery to specialised bariatric units [17], and the overall increase in patient volume which has translated into enhanced clinical outcomes [15]. Notwithstanding these advances in surgical care, the risk of mortality following bariatric surgery is far from negligible [18], in particular in cases who are considered to be ‘high risk’ through comorbidity or poor physiological reserve [19]. Enhanced recovery after surgery (ERAS) protocols are an evidence-based care improvement process for surgical patients which have been designed to negate the impact of the anticipated physiological and immunological cascade patients are subject to following major surgery [20]. ERAS protocols have been associated with reduced post-operative complications, inpatient hospitalisation duration, and hospital costs [20, 21]. Following the robust implementation and success of ERAS protocols in other surgical specialities (including thoracic [22], urological [23], and colorectal surgeries [24]), ERAS protocols have now been incorporated into the management paradigm for patients undergoing bariatric surgery, with results from observational and randomised clinical trial (RCT) data suggesting there may be benefit in postoperative outcomes expected following effective ERAS implementation [25–28]. Consequently, the ERAS Society published recommended preadmission, pre-, intra-, and post-operative ERAS guidelines in 2021 to encourage implementation and adoption of these protocols for perspective patients due to undergo bariatric surgery [29].

While previous meta-analyses have been performed and demonstrate the positive effect of ERAS protocols on outcomes following bariatric surgery [30–32], these studies are limited due to a reliance upon observational and retrospective data to decipher the potential benefit of ERAS implementation. Therefore, a meta-analysis consisting solely of RCTs is necessary to determine the value of ERAS protocols on patients undergoing bariatric surgery. Accordingly, the aim of this study was to perform a systematic review and meta-analysis of RCTs to evaluate the impact of ERAS protocols on patient outcomes following bariatric surgery.

## Methods

This systematic review was conducted in accordance with the preferred reporting items for systematic reviews and meta-analyses (PRISMA) guidelines [33]. Ethical approval was not required from the local institutional review board due to this study using data from previously published resources. All authors contributed to formulating the study protocol, and it was then registered with the International Prospective Register of Systematic Reviews (PROSPERO – CRD42023434492).

### Population, Intervention, Comparison, Outcome (PICO) Tool

Applying the PICO framework [34], the clinical research question the authors wished to address was as follows:

Population — Any patients indicated to undergo bariatric surgery in a randomised clinical trial setting.

Intervention — Any patients who were randomised to undergo ERAS.

Comparison — Any patients who were randomised to standard care (SC).

Outcomes — The study outcomes included: Overall complications, major complications, leaks, bleeding, surgical site infections (SSIs), nausea and vomiting, reoperation, intraoperative time (measured in minutes), postoperative pain, time to mobilisation, duration of intensive care unit stay (ICUS), total hospital stay (THS), functional hospital stay (FHS), 30-day readmissions, hospitalisation costs, and mortality.

### Search Strategy

An electronic search was performed of the PubMed, EMBASE and Cochrane (CENTRAL) databases on the 12th May 2023 for relevant RCTs which would be suitable for inclusion in this study. The search was performed of all fields under the following headings: (enhanced recovery after surgery) and (bariatric surgery) which were linked by the Boolean operator, ‘AND’. Included studies were limited to those published in the English language and of prospective randomised design. Studies were not restricted based on year of publication. For retrieved studies, their titles were initially screened, before the abstracts and full texts which were deemed appropriate were reviewed.

### Inclusion and Exclusion Criteria

Studies were considered for inclusion in the current study if they met the following inclusion criteria: (1) studies had to be prospective RCTs which randomised adult patients aged 18 years or older indicated to undergo bariatric surgery to ERAS or SC protocols; (2) studies had to include surgical outcomes following ERAS and SC. Studies were excluded from this study if they failed to meet the above inclusion criteria.

### Definitions

- Intensive care unit stay — time measured in hours from the end of the surgery until discharge criteria from the ICU were met.
- Functional hospital stay — time measured in hours from the end of the surgery until discharge criteria had been met, as described by Geubells et al. [28].
- Total hospital stay — time measured in hours from the end of the surgery until actual time of discharge from hospital, as described by Geubells et al. [28].
- Overall complications — all complications, as measured using the Clavien-Dindo classification for surgical complications [35]
- Major complications — complications of grade ≥ 3, as measured using the Clavien-Dindo classification for surgical complications [35]
- Postoperative pain — measured using the visual analogue scale [36]

### Data Extraction and Quality Assessment

The literature search was performed by two independent reviewers (M.G.D and N.E.D.) using a predesigned search strategy. Duplicate studies were manually removed. Each reviewer then reviewed the titles, abstracts, and/or full texts of the retrieved manuscripts to ensure all inclusion criteria was met, before extracting the following data: (1) first author name, (2) year of publication, (3) study design (including intervention and control), (4) country of research facility, (5) number of patients indicated to undergo bariatric surgery, (6) number of patients randomised to ERAS and SC protocols, and (7) surgical data (as outlined in the PICO framework). Risk of bias and methodological assessment of included studies was undertaken using the Risk of Bias 2.0 assessment for RCTs.

### Statistical Analysis

Descriptive statistics were used to determine the associations between ERAS and SC with surgical outcomes (Fisher’s exact test, †) [37]. Thereafter, outcomes for patients randomised to ERAS and SC were expressed as dichotomous or continuous outcomes, reported as odds ratios (ORs) with their corresponding 95% confidence intervals (CIs), following estimation using the Mantel–Haenszel method. Either fixed or random effects models were applied on the basis of whether significant heterogeneity (*I*^*2*^ > 50%) existed between studies included in the analysis. All tests of significance were two-tailed with *P* < 0.05 indicating statistical significance. Descriptive statistics were performed using the Statistical Package for Social Sciences (SPSS) version 26 (International Business Machines Corporation, Armonk, New York). Meta-analysis was performed using Review Manager (RevMan), Version 5.4 (Nordic Cochrane Centre, Copenhagen, Denmark).

## Results

### Literature Search and Study Characteristics

**Fig. 1:**
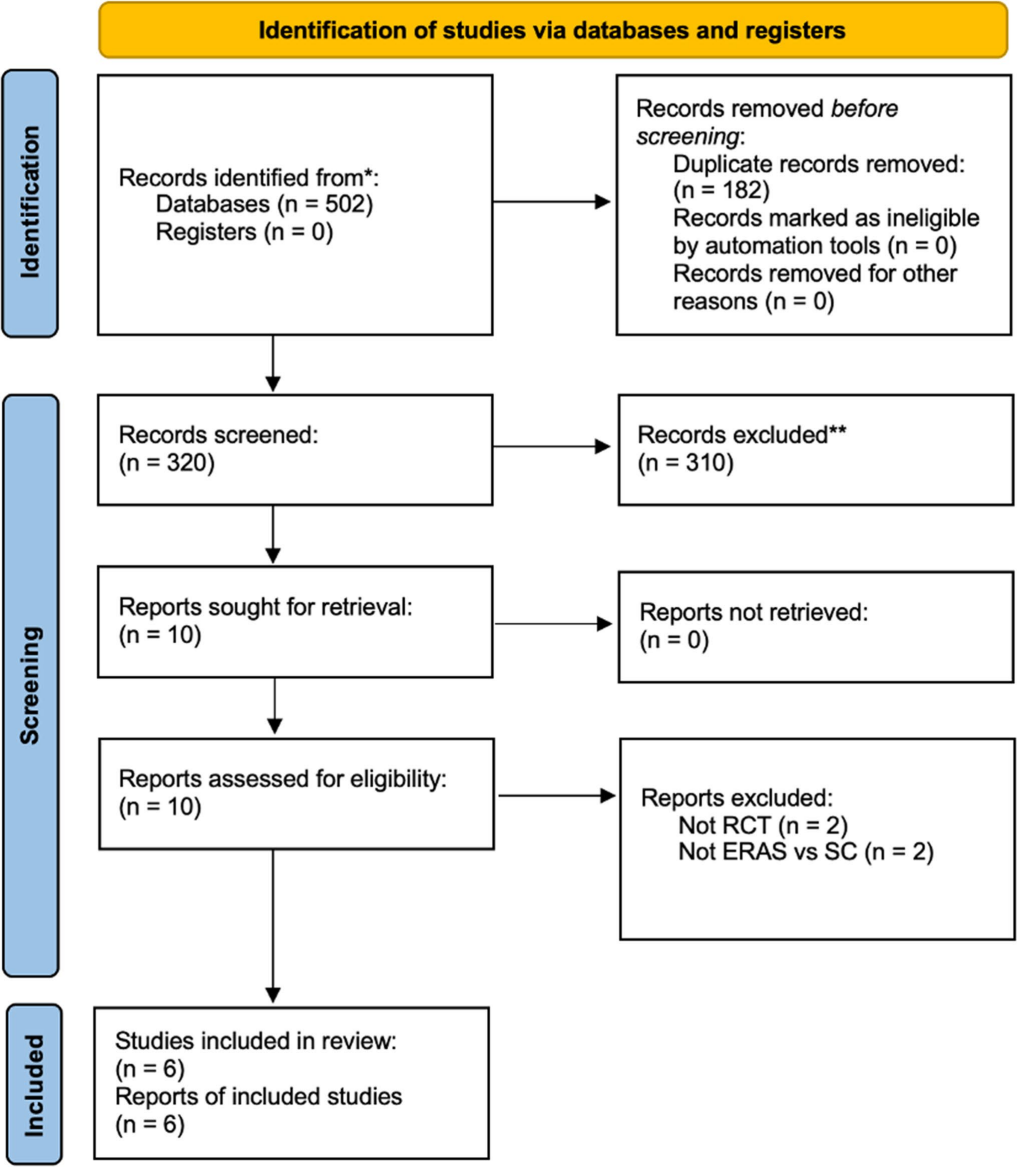
PRISMA flowchart demonstrating the systematic search process

The systematic search strategy identified a total of 502 studies, of which 182 duplicate studies were manually removed. The remaining 320 studies were screened for relevance, after which 10 had their full texts reviewed. In total, 6 RCTs met the eligibility criteria and were included in this systematic review and meta-analysis (Fig. 1). Of the 6 RCTs included in this analysis, all studies reported outcomes in relation to patients indicated to undergo bariatric surgery and who were randomised to ERAS or SC pathways (100.0%, 6/6). Publication dates of included studies ranged from 2013 to 2022. Study data, inclusion criteria, and risk of bias assessments for the 6 included RCTs are in Table 1. Breakdown of the components of each ERAS protocol from each included RCT is outlined in Table 2.

**Table 1:** Study data from the 6 included prospective, randomised clinical trials

Author	Year	Country	Study	Journal	Inclusion criteria	Surgery type	ROB 2.0
Geubbels	2018	Netherlands	RCT	BJS Open	Aged 18–65 years and their BMI was 40 kg/m2 or above or 35 kg/m2 or more plus one or more of the obesity-related co-morbidities	RYGBS	Some
Pimenta	2015	Brazil	RCT	Obesity Surgery	Aged 18 and 45 years, from both sexes, who had an initial BMI equal to or greater than 40 kg/m2	LSG	High
Lemanu	2013	New Zealand	RCT	BJS	All comers	LSG	High
Papasavas	2022	USA	RCT	Surgical Endoscopy	Adult patients between the ages of 18 and 70, who were able to read and write English	LSG	Some
Prabhakaran	2020	India	RCT	Obesity Surgery	All comers with ASA grades I-II without history of previous bariatric procedures	LSG	Some
Ruiz-Tovar	2019	Spain	RCT	SORD	-	RYGBS	Some

*ROB 2.0* risk of bias version 2.0 assessment, *RCT* randomised clinic trial, *BMI* body mass index, *RYGBS* Roux-en-y gastric bypass surgery, *LSG* laparoscopic sleeve gastrectomy, *BJS British Journal of Surgery*, *USA* United States of America, *ASA* American Society of Anesthesiologists, *SORD* Surgery for Obesity and Related Disorders

**Table 2:** Components of the enhanced recovery after surgery protocols for each randomised clinical trial included in this study

Study	Preoperative counselling	Reduce fasting times	Optimise operating schedule times	Optimise anaesthesia protocols	Multimodal analgesia	Avoidance of NGT and intra-abdominal drains	Avoidance of high IAP during leak testing	Early mobilisation
Geubbels	No	No	Yes	Yes	Yes	Yes	No	Yes
Pimenta	No	Yes	No	No	No	No	No	No
Lemanu	Yes	Yes	Yes	Yes	Yes	Yes	No	Yes
Papasavas	No	Yes	No	Yes	Yes	No	No	No
Prabhakaran	Yes	No	No	Yes	Yes	No	No	Yes
Ruiz-Tovar	Yes	Yes	No	Yes	Yes	Yes	No	Yes
	Analgesia	Anti-emetic	PPI/H2 agonists	Early enteral feeding	Rigorous glycemic control	Discharge planning	Virtual appt. day-1–2 post discharge	In-person appt. 2 weeks post discharge
Geubbels	Yes	No	No	Yes	No	Yes	No	Yes
Pimenta	Yes	Yes	No	No	No	Yes	No	No
Lemanu	Yes	Yes	No	Yes	No	Yes	Yes	Yes
Papasavas	Yes	Yes	No	Yes	Yes	No	No	No
Prabhakaran	Yes	Yes	No	No	Yes	No	No	No
Ruiz-Tovar	Yes	Yes	Yes	Yes	Yes	No	Yes	Yes

*NGT* nasogastric tube, *IAP* intra-abdominal pressure, *PPI* proton pump inhibitors, *H2* histamine-2, *appt* appointment

### Clinicopathological and Surgical Characteristics

In total, data from 740 patients was included. The mean age at diagnosis was 40.2 years. The majority of patients were female (75.6%, 517/684). The mean body weight was 122.9 kg. The mean body mass index (BMI) was 44.1 kg/m^2^. Overall, 54.1% of patients underwent Roux-en-Y gastric bypass surgery (GBS) (400/740) and 45.9% underwent sleeve gastrectomy (340/700). In total, 50.1% of patients were randomised to undergo ERAS (371/740) and 49.9% to SC (369/740). A detailed breakdown of data from included studies is outlined in Table 3.

**Table 3:** Patient data from the 6 included prospective, randomised clinical trials

Author	Total number	Number ERAS	Number other	Mean age (range)	Median body mass index	Mean body weight	Male–female
Geubbels	220	110	110	42.5 years	41.7 kg/m2	119.0 kg	28:192
Pimenta	20	10	10	35 years (25–45)	43.6 kg/m2	120.0 kg	2:18
Lemanu	78	40	38	43.7 years	46.2 kg/m2	133.8 kg	23:55
Papasavas	130	65	65	38.5 years (30–50)	43.6 kg/m2	-	24:106
Prabhakaran	112	56	56	36.4 years	44.8 kg/m2	116.0 kg	40:76
Ruiz-Tovar	180	90	90	45.1 years	44.9 kg/m2	125.6 kg	50:70
	740	371	369	40.2 years	44.1 kg/m2	122.9 kg	167:517

*ERAS* enhanced recovery after surgery, *kg* kilogrammes

### Overall Complication Rates

**Fig. 2:**
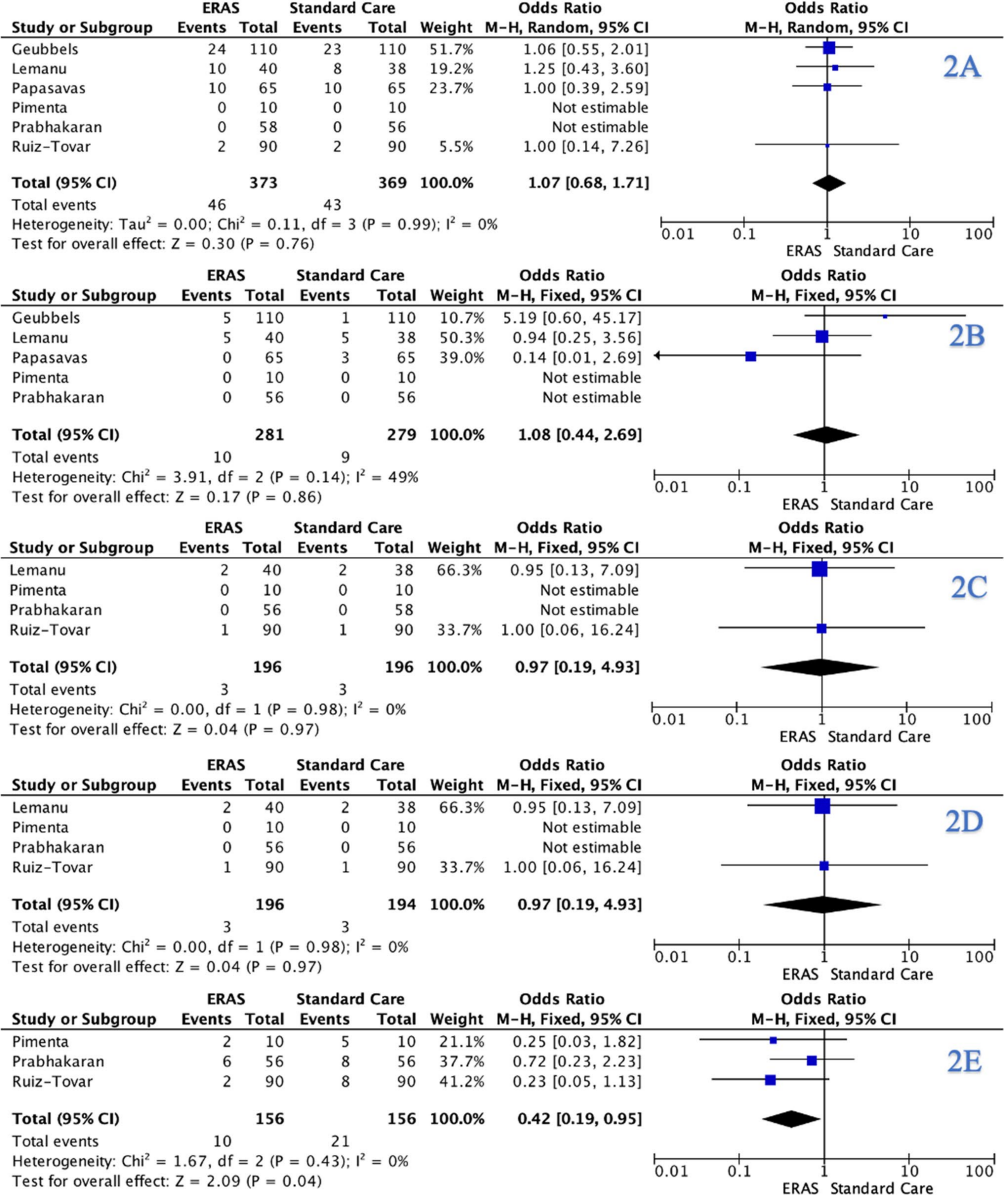
Forest plots for **A** overall complications, **B** major complications, **C** leaks, **D** bleeding, and **E** nausea and vomiting for comparison of enhanced recovery after surgery and standard care protocols following bariatric surgery

When analysing data from the 6 included RCTs, the overall complication rate was 11.8% (87/740). There was no significant difference observed in the overall complication rate for ERAS candidates compared to those who received SC (ERAS: 11.9% (44/371) vs. SC: 11.7% (43/369), *P* = 1.000, †) (Table 4). At meta-analysis, there was no significant difference observed in the overall complication rate for ERAS patients compared to those who received SC (OR: 1.07, 95% CI: 0.68–1.71, *P* = 0.760) (Fig. 2A).

**Table 4:** Descriptive statistical analysis of outcomes for patients randomised to enhanced recovery after surgery and standard care protocols after bariatric surgery

Parameter	ERAS	SC	*P*-value
Overall complications	11.9% (44/371)	11.7% (43/369)	1.000, †
Major complications	3.6% (10/281)	3.2% (9/279)	1.000, †
Anastomotic leaks	1.5% (3/196)	1.5% (3/196)	1.000, †
Bleeding	1.5% (3/196)	1.5% (3/196)	1.000, †
Nausea and vomiting	6.4% (10/156)	13.5% (21/156)	0.056, †
Reoperation rates	0.5% (1/221)	0.9% (2/221)	1.000, †
30-day readmission rates	11.9% (15/371)	11.7% (13/369)	0.848, †

† denotes Fisher’s exact test

### Major Complication Rates

In total, 5 RCTs reported major complication rates, identifying a major complication rate of 3.4% (19/560). There was no significant difference observed in the major complication rate for ERAS candidates compared to those who received SC (ERAS: 3.6% (10/281) vs. SC: 3.2% (9/279), *P* = 1.000, †) (Table 4). At meta-analysis, there was no significant difference observed in the major complication rate for ERAS candidates compared to those who received SC (OR: 1.08, 95% CI: 0.44–2.69, *P* = 0.860) (Fig. 2B).

### Leaks

Four RCTs reported leak rates. Overall, there was a leak rate of 1.5% (6/392). There was no significant difference observed in the leak rate for ERAS patients compared to those who received SC (ERAS: 1.5% (3/196) vs. SC: 1.5% (3/196), *P* = 1.000, †) (Table 4). At meta-analysis, there was no significant difference observed in the leak rate for ERAS patients compared to those who received SC (OR: 0.97, 95% CI: 0.19–4.93, *P* = 0.970) (Fig. 2C). Geubbels et al. were the only study reporting on intra-operative leak testing to assess anastomotic or staple line integrity, where methylene blue dye was used [28]. None of the included studies reported post-operative contrast swallow assessments.

### Bleeding

Four RCTs reported bleeding rates. Overall, there was a bleeding rate of 1.5% (6/390), which included 4 staple-line bleeds, 1 upper gastrointestinal bleed, and 1 splenic laceration. There was no significant difference observed in the bleeding rate for ERAS patients compared to those who received SC (ERAS: 1.5% (3/196) vs. SC: 1.5% (3/194), *P* = 1.000, †) (Table 4). At meta-analysis, there was no significant difference observed in the bleeding rate for ERAS patients compared to those who received SC (OR: 0.97, 95% CI: 0.19–4.93, *P* = 0.970) (Fig. 2D).

### Surgical Site Infection

Pimenta et al. were the only RCT that reported SSI rates. No patients developed SSIs in this study (0.0%, 0/20). Accordingly, descriptive statistical and meta-analyses were incalculable.

### Nausea and Vomiting

Three RCTs reported nausea and vomiting rates. Overall, there was an overall incidence of nausea and vomiting of 9.9% (30/312). There was a non-significant difference observed in nausea and vomiting rates for ERAS patients compared to those who received SC (ERAS: 6.4% (10/156) vs. SC: 13.5% (21/156), *P* = 0.056, †) (Table 4). At meta-analysis, there was a significant difference observed in the nausea and vomiting rates for ERAS patients compared to those who received SC (OR: 0.42, 95% CI: 0.19–0.95, *P* = 0.040) (Fig. 2E).

### Reoperation Rates

**Fig. 3:**
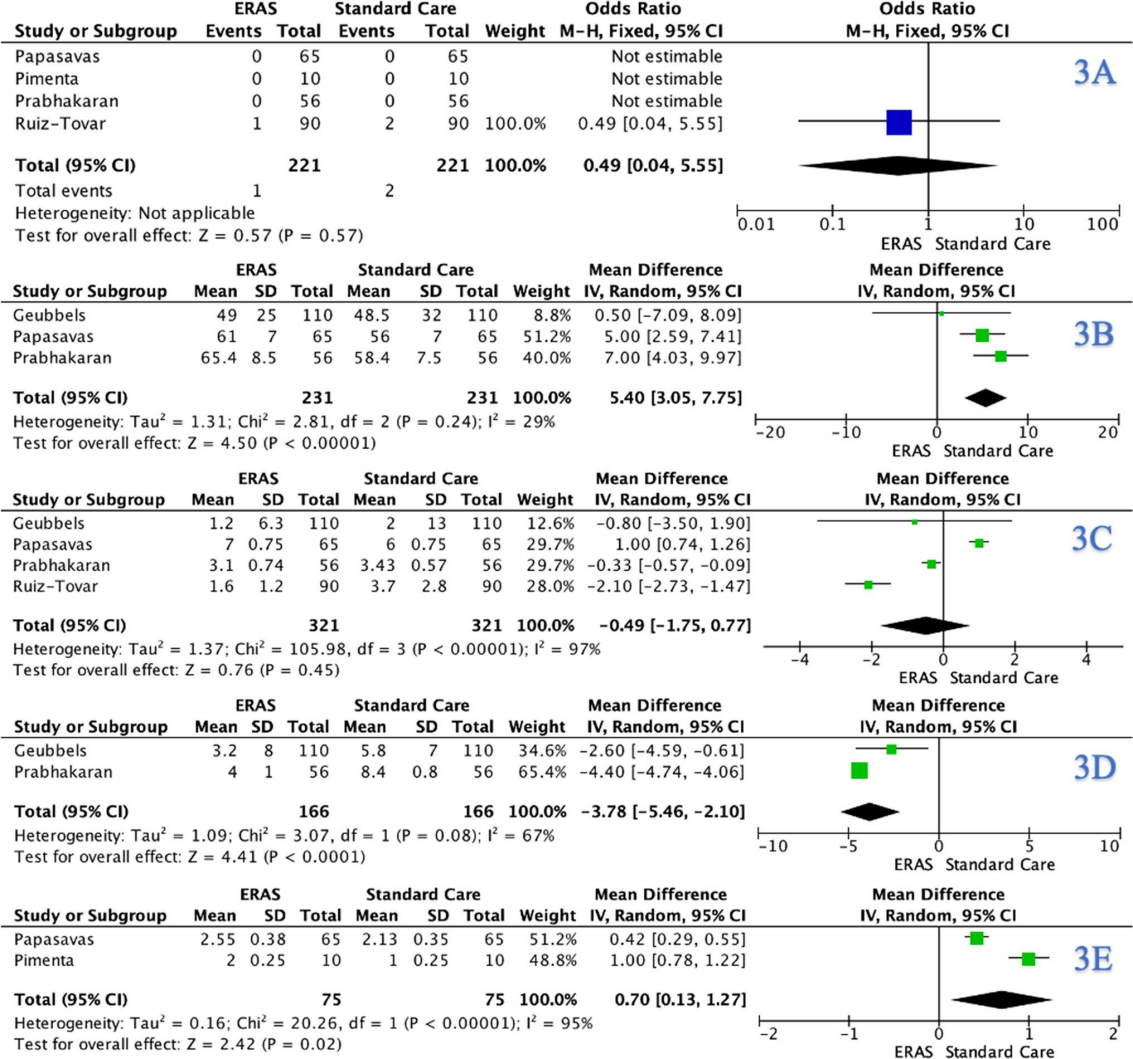
Forest plots for **A** reoperation rates, **B** intraoperative time, **C** postoperative pain using the visual analogue scale, **D** time to mobilisation, and **E** intensive care unit stay time for comparison of enhanced recovery after surgery and standard care protocols following bariatric surgery

In total, 4 RCTs reported results in relation to reoperation, which demonstrated a reoperation rate of 0.7% (3/442). There was no significant difference observed in reoperation rates for ERAS patients compared to those who received SC (ERAS: 0.5% (1/221) vs. SC: 0.9% (2/221), *P* = 1.000, †) (Table 4). At meta-analysis, there was no significant difference observed in the reoperation rate for ERAS patients compared to those who received SC (OR: 0.49, 95% CI: 0.04–5.55, *P* = 0.570) (Fig. 3A).

### Intraoperative Time

Three RCTs reported intraoperative time for patients subject to ERAS and SC protocols. At meta-analysis, there was significant increased observed in the intraoperative time for SC patients compared to ERAS (MD: 5.40, 95% CI: 3.05–7.77, *P* < 0.001) (Fig. 3B).

### Postoperative Pain

Four RCTs reported postoperative pain using the visual analogue scale (VAS). At meta-analysis, there was a non-significant difference observed in postoperative pain for ERAS patients compared to those who received SC (MD: − 0.49, 95% CI: − 1.75–0.77, *P* = 0.450) (Fig. 3C).

### Time to Mobilisation

Two RCTs reported time to mobilisation for patients subject to ERAS and SC protocols. At meta-analysis, there was significant reduction in time to mobilisation for ERAS patients compared to SC (MD: − 3.78, 95% CI: − 5.46 to − 2.10, *P* < 0.001) **(**Fig. 3D).

### Intensive Care Unit Stay

Two RCTs reported outcomes in relation to ICUS. At meta-analysis, there was significant reduction in ICUS times for ERAS patients compared to SC (MD: 0.70, 95% CI: 0.13–1.27, *P* = 0.020) (Fig. 3E).

### Total Hospital Stay

**Fig. 4:**
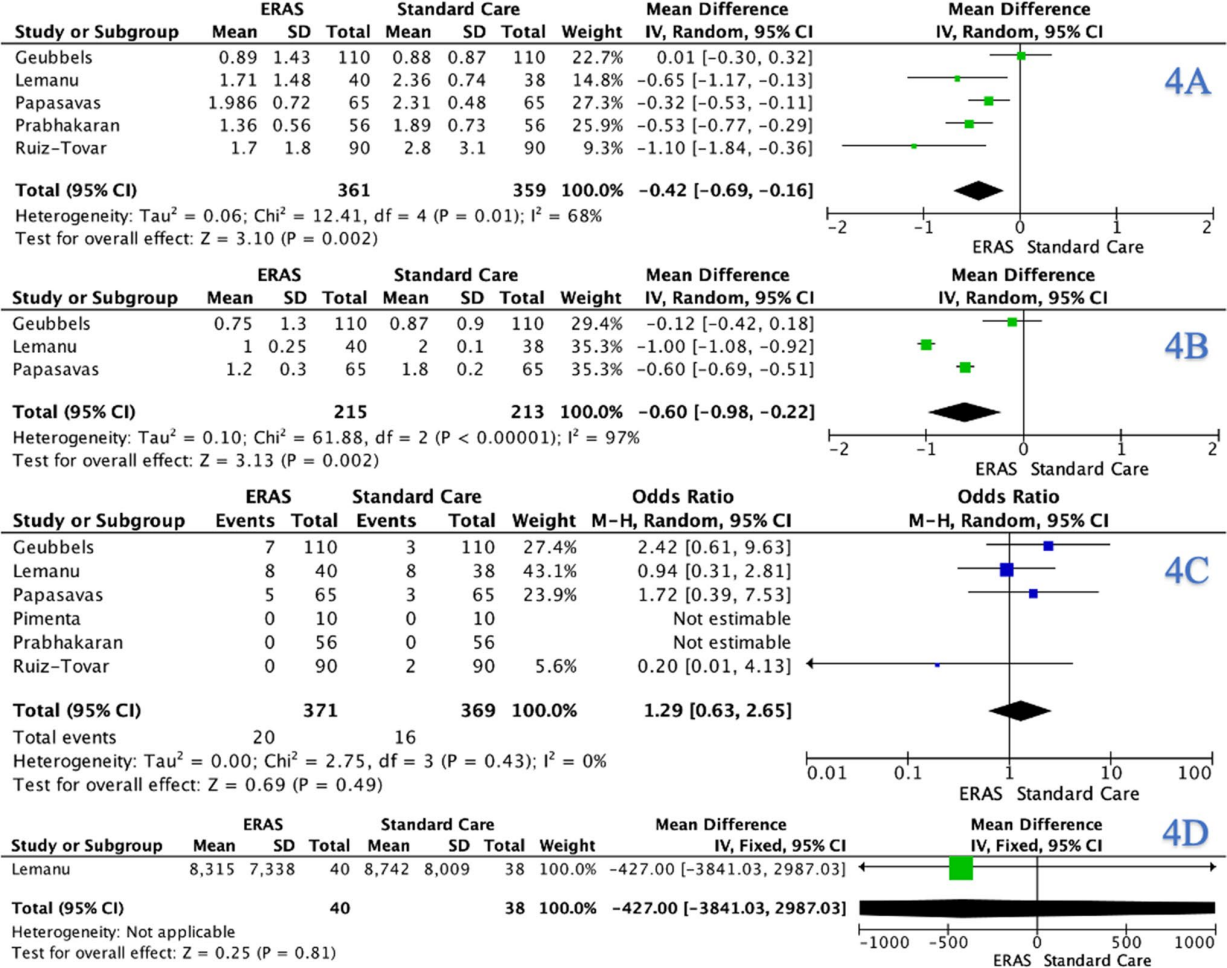
Forest plots for **A** total hospital stay, **B** functional hospital stay, **C** 30-day readmission rates, and **D** hospitalisation costs for comparison of enhanced recovery after surgery and standard care protocols following bariatric surgery

Five RCTs reported outcomes in relation to THS. At meta-analysis, there was a significant reduction in THS for ERAS patients compared to those who received SC (MD: − 0.42, 95% CI: − 0.69 to − 0.16, *P* = 0.002) (Fig. 4A).

### Functional Hospital Stay

Three RCTs reported outcomes in relation to FHS. At meta-analysis, there was a significant reduction in FHS for ERAS patients compared to those who received SC (MD: − 0.60, 95% CI: − 0.98 to − 0.22, *P* = 0.002) (Fig. 4B).

### 30-Day Readmissions

All 6 RCTs reported 30-day readmission rates. Overall, there was a 30-day readmission rate of 3.8% (28/740). There was no significant difference observed in the 30-day readmission rate for those ERAS patients compared to SC (ERAS: 11.9% (15/371) vs. SC: 11.7% (13/369), *P* = 0.848, †) (Table 4). At meta-analysis, there was no significant difference observed in the 30-day readmission rate for ERAS patients compared to SC (OR: 1.29, 95% CI: 0.63–2.65, *P* = 0.490) (Fig. 4C).

### Hospitalisation Costs

Lemanu et al. were the only RCT reporting hospitalisation costs. At meta-analysis, there was a non-significant reduction in costs for ERAS patients compared to those who received SC (MD: − 427.00, 95% CI: − 3841.03–2987.03, *P* = 0.810) (Fig. 4D).

### Mortality Rates

Four of the included RCTs reported mortality rates. No patients died during these studies (0.0%, 0/740). Accordingly, descriptive statistical and meta-analyses were incalculable.

## Discussion

This systematic review and meta-analysis integrated data from six prospective, randomised studies which evaluated the impact of ERAS protocols on both peri- and postoperative clinical outcomes following bariatric surgery. Overall, 740 patients (vastly representative of real-world patients undergoing bariatric surgery) were included, and the results from this meta-analyses demonstrate favourable clinical outcomes for those who were randomised to ERAS protocols (compared to SC) when undergoing bariatric surgery. While these data support the results of previous reviews [30, 32, 38], this study provides novelty through combining the highest possible quality evidence to demonstrate the significant benefits which may be anticipated through robust ERAS adherence when performing bariatric surgery. Moreover, this review incorporates recent data from bariatric units from all over the world, providing a congruent message in support of using ERAS protocols as a strategy to improve outcomes, irrespective of cultural exposure, healthcare (in)equity, or the genetic composition of the local population in question. Thus, this data supports implementation of ERAS protocols for suitable candidate patients indicated to undergo bariatric surgery, where feasible.

As outlined, these results support the routine utility of ERAS protocols in primary bariatric surgery where feasible. This is due to a significant reduction being observed in the proportion of patients experiencing nausea and vomiting, coupled with significant reduction in intraoperative duration, time to mobilisation, ICUS, THS, and FHS, while not increasing complications or readmission rates as a consequence. In tandem, with reduced hospital and ICU stays, these results suggest positive implications for patient health, quality of life metrics, and clinical outcomes following bariatric surgery [39]. Thus, these results demonstrate the potential cost effectiveness of using ERAS protocols in day-to-day clinical practice, albeit not directly demonstrated in the results of this rudimentary systematic review. In essence, reduced intra-operative time, ICUS, THS, and FHS will have positive implications on the cost-effectiveness of the provision of bariatric services are significant when attempting to optimise healthcare economies across the globe, particularly as we move towards ‘fast track’ and’23-h’ hospital discharges following bariatric surgery [40, 41]. For example, several previous high-quality studies have illustrated the cost-effectiveness of ERAS in other surgical specialties, including those reporting outcomes following hepatobiliary [42], colorectal [24], and thoracic resections [22], which is secondary to an overall reduction in patient morbidity and hospital stay, when ERAS has been diligently implemented and adhered to [43, 44]. Furthermore, if such measures were adopted in the setting of high-volume bariatric-dedicated high dependency units, costs could theoretically be further extenuated through increased efficiency and experience in managing these patients post-operatively [45]. Albeit this cost-effectiveness was not directly observed in the results in the current review, this shortcoming is most plausibly explained by a type II statistical error [46], which is due to results from just 1 RCT (where *n* = 78) being integrated and analysed for this outcomes measure. Therefore, it is plausible that ERAS following bariatric surgery has similar cost-effectiveness to that noted in other surgical specialties [22, 24, 42], as has been previously demonstrated in observational studies in bariatric surgery [47].

Traditionally, enhancing surgical outcomes focused primarily upon advancing technological innovations, the centralisation of specialised (complex or revisional) cases, and increasing the adoption of checklists to ensure the provision of consistently high-quality care [48–50]. These timely changes have improved patient outcomes; however, providing benchmark care to the increasing numbers of people living with obesity creates several surgical and anaesthetic challenges, which must be combatted by pre-empting the physiological and immunogenic sequelae that follow surgical manipulation of the upper gastrointestinal tract. Importantly, the data explored in this meta-analysis of RCTs supports the pragmatism of implementing ERAS protocols in this unique and increasingly more common patient cohort, though the illustration of promise these simple adjustments have in further optimising patients prior to and during bariatric surgery.

Despite several strengths, the authors acknowledge that this study is subject to several unavoidable limitations. Firstly, as stands true for the majority of RCTs performed in the field of surgery, none of the included RCTs in this analysis were ‘blinded’. The inability to blind surgeons to interventions leads to these RCTs to be classed as ‘open label’, making them subject to unintentional biases [51]. Secondly, it is plausible that certain potential confounders (for example, patient age, patient comorbidities, surgeons experience) may influence the results of this study, and the authors also acknowledge the measurement of the severity of morbidities and clinical conditions triggering readmission are not measured in the current analysis. Thirdly, none of the included studies provided data surrounding patient reported perspectives in relation to ERAS protocols following bariatric surgery, thus limiting subjective insights this study provides into the impact of ERAS on the patient experience of bariatric surgery. Finally, while data was compiled from all available RCTs published on this topic, this meta-analysis relies on data from just 740 patients, limiting the robustness of results. Despite these limitations, this meta-analysis of RCTs provides comprehensive, high-quality analyses which support the implementation of ERAS protocols to bariatric surgical practice, where feasible.

In conclusion, this systematic review and meta-analysis of RCT data demonstrates the clinical utility of ERAS protocols in reducing post-operative nausea and vomiting, time to ambulation, ICUS, FHS, and THS. Based on the results of this study, we advocate for the routine implementation of ERAS protocols to bariatric surgical units, where feasible. Thus, the next generation of prospective randomised clinical trials should focus on refining and adjusting our approach to ERAS following bariatric surgery, in order to further improve outcomes for patients undergoing bariatric surgery.

## Data Availability

Data will be made available upon reasonable request from the corresponding author.
